# Gender-Based Heat Map Images of Campus Walking Settings: A Reflection of Lived Experience

**DOI:** 10.1089/vio.2023.0027

**Published:** 2024-03-11

**Authors:** Robert A. Chaney, Alyssa Baer, L. Ida Tovar

**Affiliations:** ^1^ Department of Public Health, Brigham Young University, Provo, Utah, USA.; ^2^ Milken Institute School of Public Health, The George Washington University, Washington, District of Columbia, USA.; ^3^ Division of Public Health, University of Utah School of Medicine, Salt Lake City, Utah, USA.

**Keywords:** fear of crime, gender, safety, walking, college health, heat map images

## Abstract

Fear of crime can influence our view of and experience with the world around us. This can be problematic for individuals seeking physical activity, including from walk commuting. Prior work shows fear is especially evident among women, who report fear of rape and sexual abuse by men as a primary concern. We present the results of a cross-sectional survey (*n* = 571) where participants were shown images from college campus (*n* = 4 campuses) depicting different lighting (daytime, nighttime), and entrapment levels (high, low; i.e., able to easily escape if needed, with high entrapment being difficult and low being easy), and using the Qualtrics heat map tool, selected features that stood out to them most. Data were segregated by gender and analyzed to determine similarity of heat maps for the same base image. Heat map images were analyzed using canonical correlation (*Rc*) to determine the relationship between the two groups; dispersion testing to decipher spatial uniformity within the images; the Structural Similarity Index (SSIM) to characterize the nature of image patterns differences; and, the Breslow–Day Test to specify pattern locations within images. Several heat map images are also presented in the results. Overall, female and male participants appear to “see” different things when imagining walk-commuting (as seen by poor *Rc* values) and the nature of what they were looking at were different (as seen by poor SSIM values). Female participants tended to focus on areas outside the walking path, such as bushes and dark areas, whereas men's focus was on the path ahead [*χ*^2^(1) = 4.29, *p* = 0.04]. Furthermore, women were more likely to select areas outside the walking path during high entrapment settings [*χ*^2^(1) = 15.49, *p* < 0.001] and at nighttime [*χ*^2^(1) = 4.98, *p* = 0.02]. Our study demonstrates point-of-view differences in female–male walking space assessments. Viewing walking safety through the lens of lived experience could be productive for holistic community walking safety.

## Introduction

Our view of and experience with the world can have far-reaching influence on our health, both in the short and long run. For example, fear can decrease motivation for physical activity (Foster et al., 2014a; Stodolska et al., 2013). Multiply this over the course of one's college experience, or adult life, and the results are troubling (Poobalan et al., 2012; Telama et al., 2005). At times, fear unfairly burdens those it afflicts by limiting their power to pursue life, liberty, and happiness (Kennedy, 2023; Rierson, 1994). Most research about female college student nighttime commuter safety has been set in European countries, although there is a growing body of research based in North America. Time of day appears important in determining feelings of safety and risk for crime. Sunset conditions increase perceptions of danger and pose greater risk for women who commute or exercise at night (Boomsma and Steg, 2014). Fear associated with nighttime, or poorly lit, commuting has been shown to negatively impact female students' physical, emotional, and social health (Burdette and Needham, 2012; Larson et al., 2013; Park and Garcia, 2020). In addition, research shows safety perceptions can greatly impact sleep patterns and academic performance (Etopio et al., 2019).

Bolstering community walking conditions around college campuses is imperative owing to the link between freedom to move around a community and basic health outcomes (Boomsma and Steg, 2014; Peachey and Baller, 2015). Gaining an understanding of the factors that affect female college students' safety can give evidence to drive community improvements within the college community. By recognizing and assessing the influence of the built environment and safety perceptions on female college students walking autonomy, we add information about needed environmental and systems changes within U.S. college towns to promote improved female college student health and wellbeing.

Dutch university researchers, Boomsma and Steg, considered the interplay of physical environment, that is, lighting and entrapment, and individual factors, such as gender, on feelings of safety (2014). The available research about student safety perceptions has predominately been conducted in European settings and the transferability to U.S. college communities is not known. In this study, we differentiate heat map patterns, heat maps being a visual representation of data across a geographic or image space, between male and female participants when viewing the same college campus image. This approach builds upon the methodological foundation they developed, namely entrapment (ability to easily escape if needed) and lighting, but we displayed images from several different college campuses and differentiated heat maps by gender (Boomsma and Steg, 2014).

## Background

Current research shows a relationship between the built environment, individuals' perception of safety, and their subsequent behaviors (Blöbaum and Hunecke, 2005; Petherick, 2000). To assess these relationships, two prominent physical environmental studies used methods to expose participants to different scenarios to determine gender-based perceptions of lighting and entrapment levels among students (Blöbaum and Hunecke, 2005; Boomsma and Steg, 2014). Overall, results indicated participants felt safer in low entrapment and higher lighting settings (Boomsma and Steg, 2014) and that participants felt most uneasy in areas with low lighting, high entrapment, and high concealment because of the perception that poorly lit areas are hiding spots for potential assailants (Blöbaum and Hunecke, 2005).

Others have noted that individual's feeling of safety, particularly students, is related to these features in the physical environment, notably lighting and entrapment, and that these perceptions can lead to changes in commuting behavior, by way of changing routes to avoid places that were poorly lit or had a reputation for being dangerous (Boomsma and Steg, 2014; Foster et al., 2014b; Loukaitou-Sideris and Eck, 2007; Mason et al., 2013; Petherick, 2000). These findings are consistent with the theory of “prospect and refuge,” which shows that individuals prefer environments where they are able to observe (prospect) without being observed (refuge) (Fisher and Nasar, 1992).

Research identifies gender itself as one of “strongest predictors” of feeling safe against human actions in public spaces (Blöbaum and Hunecke, 2005; Boomsma and Steg, 2014; Loewen et al., 1993). In multiple studies, women report higher rates of fear than their male counterparts (Fisher and Sloan, 2003; Valentine, 1989). Women were also more likely to demonstrate avoidance behaviors and generate “mental-maps,” which could be based on past experience or secondary information, to avoid locations based on personal experience or anecdotal evidence of higher risk locations (Petherick, 2000; Valentine, 1989). According to some, the factors that are considered primary reasons for this perception disparity include both social messaging of gender roles and fear of rape crime—which has been cited as the highest feared crime by women (Fisher and Sloan, 2003; Valentine, 1989).

There is good reason for this trepidation. Women aged 18–24 years old are four times more likely to experience sexual violence (three times more for 18–24 year old women who are in college) than women of other age groups (RAINN: Rape, Abuse and Incest National Network, 2023a). In fact, 26.4% of all undergraduate women experienced rape or sexual assault during college, although these numbers may be deflated by underreporting (Jones, 2009; RAINN: Rape, Abuse and Incest National Network, 2023a; Taylor, 2018). To illustrate this occurrence, we will compare robbery and sexual assault. In the general population, there are roughly 1.25 robberies for every 1 sexual assault; among college women, there are 2 sexual assaults for every 1 robbery. Only 19.5% of rapes are committed by a stranger to the victim (this percentage is lower for children and teens, 7%), so this feeling of being “on-guard” appears to travel with the person despite changes in risk (RAINN: Rape, Abuse and Incest National Network, 2023b).

Female college students who feel unsafe commuting at night experience a negative impact on their physical, emotional, and social health (Burdette and Needham, 2012; Kirk, 1988; Larson et al., 2013). Safety perceptions can impact sleeping patterns, academic performance, and mental health (Boomsma and Steg, 2014; Etopio et al., 2019). Poor perceived safety has been linked to higher BMI as well as a decreased regular physical activity (Burdette and Needham, 2012; Larson et al., 2013; Peachey and Baller, 2015;Turner et al., 2017). Stressful situations, like fear, can also produce internal changes and have been associated with higher levels of cortisol, elevated heart rate, and perspiration (Hakamata et al., 2017; Thau et al., 2023; Zhang et al., 2019).

These physiological responses directly related to perceptions of control and safety have been shown to increase worry—defined as “a chain of repetitive thoughts that is experienced as relatively uncontrollable” (Steinfurth et al., 2017). This is concerning because college years are formative for adulthood; attitudes and behaviors developed during this time often continue into adulthood (Burdette and Needham, 2012; Larson et al., 2013; Peachey and Baller, 2015; Turner et al., 2017).

There are important gaps in current literature about the experiences of college student walkers: perceptions of safety and physiological manifestations of perceived safety. This is especially evident at U.S. campuses. We approach this subject with a critical geography lens, including the spatial and social constraints of women's travel (Kwan, 2000). It is known that men and women use shared spaces and commuting modes differently, partly from fear-based differences and to some degree to gender role differences (Kwan, 2000; Ravensbergen et al., 2019). This lends to our lives, women's included, being shaped by the social and physical environments we live in (Preston and Ustundag, 2005). Beebeejaun further argues that claiming everyday lived spaces is vital to an individual's entitled rights to mobility freedom within a community (2017).

Our work provides insight into differences in lived experience by showing what they see. We sought to differentiate heat map patterns between male and female participants when viewing the same college campus image. More specifically: (1) How statistically related are male–female heat map patterns looking at the same image?, (2) Do clustering patterns exist within the images (suggesting a focus of attention in the image)?, (3) If clustering exists, are they inherently different between genders?, and (4) If clustering exists, are there differences in where clusters are located with respect to physical features in the images (i.e., walking path)?

## Methods

### Data acquisition

Adult college students at Brigham Young University (BYU) were recruited during a 2-week period in spring, 2021 using social media platforms and groups popular among this population, for example, Facebook and Instagram accounts for the department and college, and several campus clubs with diverse membership (Whitaker et al., 2017). A 69-item online Qualtrics survey explored student views on walk-commuting and safety through different campus environments and was approved by the university Institutional Review Board. Participants were given 16 images and asked to consider walking alone through the place in the picture. Using the Qualtrics heat map tool, they were instructed to imagine themselves walking through these areas and to click on the area(s) of the image that stood out to the most to them.

**FIG. 1.**
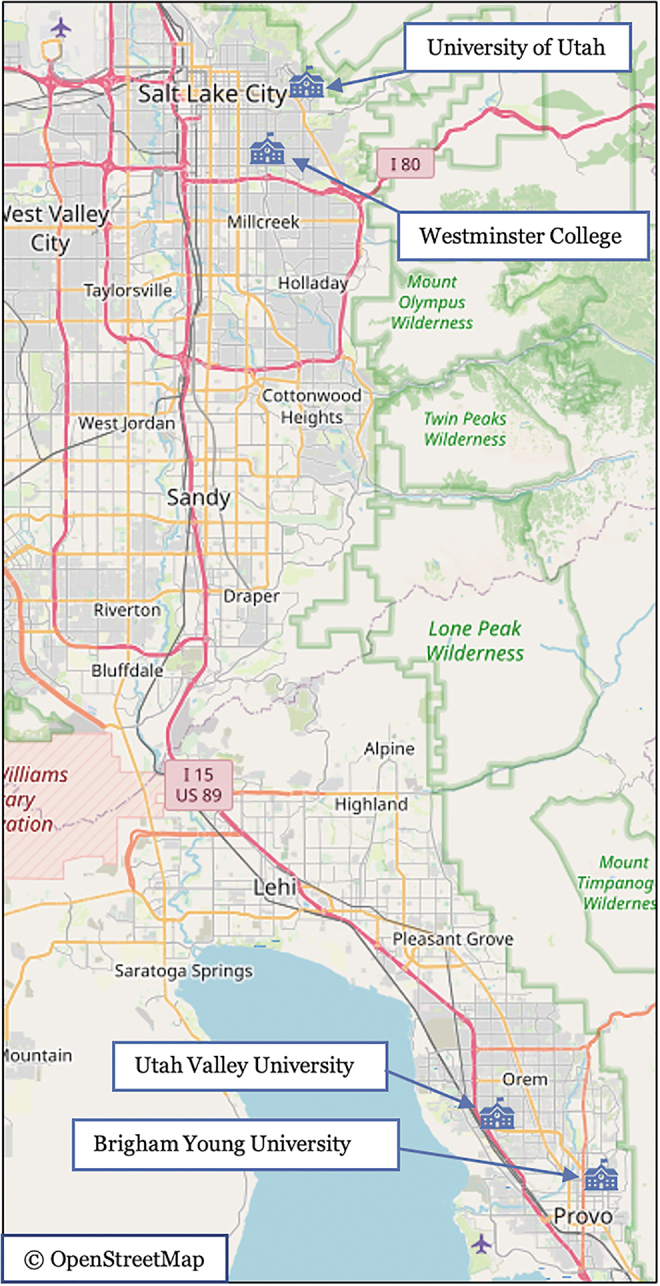
Study Image Locations in North-central Utah. Image obtained from OpenStreetMap under the Open Database License (OpenStreetMap contributors, 2023).

All pictures represented the 2 × 2 × 4 factors: two entrapment levels (high, low), two time of day levels (daytime, nighttime), and four campuses. These campuses are located within 40 miles of each other (65 km) in north-central Utah: University of Utah (UU), BYU, Westminster College (WC), and Utah Valley University (UVU) (Fig. 1). Over two-thirds of BYU students are from out of state and we assume they are generally unfamiliar with these other campuses. The specific locations chosen were chosen based on their entrapment characteristics and not necessarily if that location was “notable.”

### Data analysis

For statistical analysis of the heat map images, each image was treated as an information containing matrix, as this is all a digital image anyway, a collection of pixels or “cells” representing red-blue-green color or grayness in the case of black and white images (Mazet, 2023; Nashville Film Institute, 2022). All data were analyzed using R statistical software 4.2.2 (R Core Team, 2022), the *spatstat* (v3.0.2; Baddeley and Turner, 2005), *SpatialPack* (v0.4; Vallejos et al., 2020), and *DescTools* (v0.99.47; Signorell et al., 2022) packages. The relationship between these image matrices was determined using canonical correlation (*Rc*) (Andrew et al., 2013; Borga, 2001). This is the multivariate version of the more familiar univariate, Pearson's correlation (*r*) and is interpreted similarly.

Instead of examining the relationship between two columns of data, *Rc* determines the relationship between two matrices of data. Imagine taking two semi-transparent n × n matrices of the same size and placing one on top of the other, you could see where similarities and differences are; *Rc* assesses the observed overlaps statistically.

To avoid confirmation bias, or the “rage to conclude” as Tufte (2006) puts it, we sought to verify observed patterns. The nature of each image's general point pattern, whether it was statistically clustered or not, was determined using the dispersion test for spatial point patterns. In essence, it performs a chi-squared test for homogeneity on the given matrix, determining if the observations are uniform across the matrix, or if there is statistically significant clustering (Baddeley and Turner, 2005; Cressie and Read, 1984). Knowing the existence of statistical clusters naturally leads to seeking to discern between those clusters globally and locally, that is, are these clusters arranged in different places in the images between genders and if so, where at? The nature of the image clusters was examined using the Structural Similarity Index (SSIM) (Horé and Ziou, 2010).

This index compares two images, *SSIM = L·C·S,* where L = luminance, or brightness; C = contrast, or brightness range, and S = structural composition (where objects are located within the image itself); holding *L* and *C* constant between the images, as in our case they are using identical background give insight into differences in the image's *S*, structure. The resulting SSIM value is bound by 0 and 1, where 0 is completely dissimilar images and 1 are exact matches. Determining differences in cluster location across settings and between genders (i.e., if points were located inside the boundaries of the walking path or not) were determined using the chi-squared test for independence and Breslow–Day test for homogeneity, a multivariate version of the chi-squared test. For example, the *χ*^2^ allows for gender × path, whereas the Breslow–Day allows for gender (M/F) × path (in/out) × entrapment level (high/low).

## Results

A total of *n* = 571 respondents completed the survey; *n* = 276 (55.8%) were female, *n* = 219 (44.2%) were male, *n* = 71 were nonbinary (12.4%), roughly matching the university demographics profile (51% female, 49% male, 81% white) (Brigham Young University, 2022). Although the sample included responses received from nonbinary students, the stratified sample sizes were low for further breakdowns, that is, critical mass of points within images or for contingency analysis was too small (*post hoc* power analysis confirmed). For the purpose of assessing our research questions, only male and female participant data have been included, as was the case in Boomsma and Steg (2014).

### Relationship of images

In general, male to female images were poorly related to each other, as shown using canonical correlation, *Rc*. The low entrapment × daytime × BYU campus image showed the strongest relationship (i.e., less difference) between males and females (*Rc* = 0.56). Conversely, the low entrapment × nighttime × WC image showed the weakest relationship (*Rc* = 0.24), suggesting greater male–female difference between images.

### Existence of clusters in images

All images showed significant point clustering based on dispersion test for spatial point patterns (all *p* < 0.001). Now that clusters were detected across all images, our focus shifted to identifying their shapes and locations, thus signifying possible differences in focus.

### Nature of the clusters

**FIG. 2.**
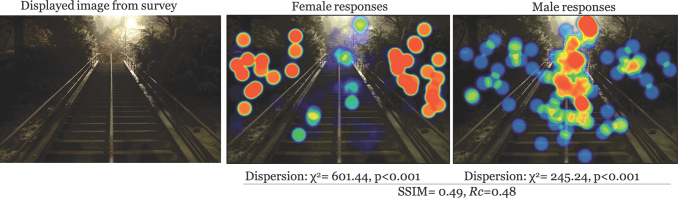
Comparison example: High entrapment × nighttime × BYU. BYU, Brigham Young University.

**FIG. 3.**
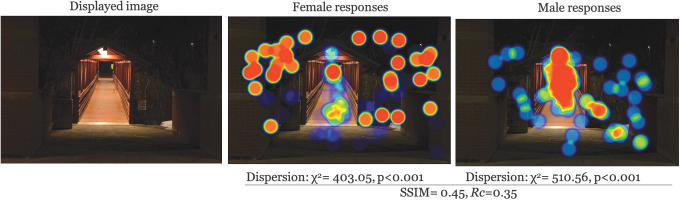
Comparison example: High entrapment × nighttime × WC. WC, Westminster College.

**FIG. 4.**
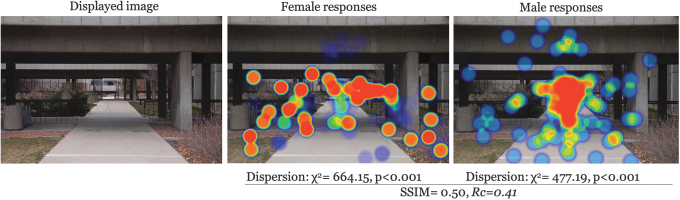
Comparison example: High entrapment × daytime × UU. UU, University of Utah.

**FIG. 5.**
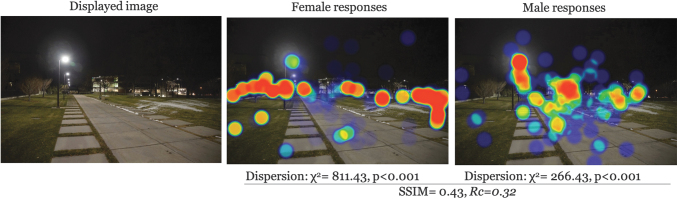
Comparison example: Low entrapment × nighttime × UVU. UVU, Utah Valley University.

**FIG. 6.**
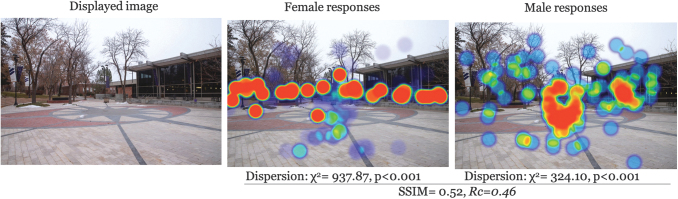
Comparison example: Low entrapment × daytime × WC.

There was moderate to low similarity in the structure of clusters across all images (avg SSIM = 0.47). The most similar images participants were for low entrapment × daytime × UU (SSIM = 0.55); furthermore, high entrapment × nighttime × UU was the lowest similarity (SSIM = 0.42) (Table 1 for all comparisons). This, in addition to the *Rc* for each image suggests a systematic difference between genders in what caught their attention in image observations. A graphical representation makes this point clearer. Examples for each entrapment × time of day scenario are given in Figures 2–6. Each displays the original campus image shown to participants (left), the female responses (center), and male responses (right). The images, across different conditions, not only seemed to show a systematic difference between female and male participants, but what they were focusing on appeared different. This naturally led to examining if there were a statistical difference in the location of foci between gender groups.

**Table 1.** Image Comparisons Between Male–Female Genders Across Physical Environmental Settings and Campuses

Campus	Time of day	Entrapment	SSIM	Rc
BYU	Day	High	0.43	0.55
		Low	0.53	0.56
	Night	High^a^	0.49	0.48
		Low	0.44	0.42
UU	Day	High^a^	0.50	0.41
		Low	0.55	0.48
	Night	High	0.42	0.35
		Low	0.45	0.32
UVU	Day	High	0.50	0.41
		Low	0.46	0.48
	Night	High	0.49	0.35
		Low^a^	0.43	0.32
WM	Day	High	0.52	0.48
		Low^a^	0.52	0.46
	Night	High^a^	0.45	0.35
		Low	0.45	0.24

^a^ Example given in Figures.

BYU, Brigham Young University; SSIM, Structural Similarity Index; UU, University of Utah; UVU, Utah Valley University; WM, Westminster College.

### Location of focus

Categorical analysis focused on points being inside or outside of the walking pathway in the images (for example, see Figure 1). The distribution of points, whether in the path or out, was inhomogeneous across all campuses [*χ*^2^(3) = 83.72, *p* < 0.001] (Table 2). Both BYU and Westminster had more outside of path selections than inside. There was an inhomogeneous relationship between path selection and entrapment level [*χ*^2^(1) = 221.58, *p* < 0.001]. There were many more female responses selecting out of path regardless of condition compared with men [*χ*^2^(1) = 4.29, *p* = 0.04].

**Table 2.** Categorical Analysis of Participant Point of Focus Within Heatmap Images

Conditions	χ^2^ (df)	*p*
Campus × path	83.72 (3)	<0.001
Time of day × path	0.85 (1)	0.36
Gender × path	221.58 (1)	<0.001
Entrapment × path	4.18 (1)	0.04
Gender × path × time of day^a^	4.98 (1)	0.02
Gender × path × campus^a^	7.18 (3)	0.07
Gender × path × entrapment^a^	15.49 (1)	<0.001

^a^ Breslow–Day test of homogeneity.

The Breslow–Day test examined the gender × path relationship within context of a third dimension. Path selection proved different across gender × time of day [*χ*^2^(1) = 4.98, *p* = 0.02], although it was homogeneous when only examining the two dimensions time of day × path [*χ*^2^(1) = 0.85, *p* = 0.36]. And path selection was different when looking at gender × entrapment [*χ*^2^(1) = 15.49, *p* < 0.001]. Path × entrapment showed a dependent relationship in the two-dimensional case [*χ*^2^(1) = 4.28, *p* < 0.04], but in the three-dimensional case, when adding the dimension of gender, more clearly parses out the difference.

## Discussion

Male and female responses demonstrated they were seeing different things despite being shown the same images. This was evident by stark gender-based differences in viewing hotspot images and with supporting statistical analysis. This finding supports the findings of Boomsma and Steg that men and women experience their built environments differently (2014). For the most part, male respondents tended to identify paths and walkways, whereas female respondents were more likely to point out areas outside the path, such as bushes or dark areas. This was especially true at night and in high entrapment areas. This suggests that because the environment is perceived and experienced differently by women and men, decisions in building campus environments should consider the varied experiences, perceptions, and safety of both as well. The built environment, however, is not the sole producer of sense of safety and other social and cultural factors should also be considered, including the systems from which these environments are made (Preston and Ustundag, 2005).

Gender-based differences in sense of safety (or fear of crime) has been documented (Blöbaum and Hunecke, 2005; Boomsma and Steg, 2014). It is likely women are more aware of potential crimes (Wilson and Little, 2008). And this fear is likely carried around with them despite changes in actual risk, for example, fear of crime, particularly sexual assault, while walking where the risk is much higher to be perpetrated by someone they know (RAINN: Rape, Abuse and Incest National Network, 2023b). The findings presented here provide a glimpse through the eyes of different walkers. These point-of-view images give some insight to the scope of what it is like to walk home as a woman—which could be multiplied through years, or a lifetime, of experiences in their built environment.

These findings can be useful for viewing campus safety, particularly walking, more holistically. Many campuses use on-call safety apps where walkers can press a button for emergency circumstances. This reactionary approach ignores the lived experience and long-term impact of fear for a woman walking home, and as seen, the way that this typically differs from the experiences of men. An app is not going to fix that. The same argument holds for lighting. Although lighting is a critical part of built environments, the heat maps demonstrate that, even where there were lighted paths (e.g., Figs. 2–3), women still looked outside the path, suggesting a more systematic problem in the way men and women interact with the built environment (Kwan, 2000; Ravensbergen et al., 2019). While lighting remains critical in terms of deterring criminals (Welsh and Farrington, 2008), its impact in improving wellbeing is unknown.

Results here should be viewed in the light of the study limitations. Data were collected from a single college student population, at one point in time, during the COVID-19 pandemic, and campus images only represented colleges and universities in confined geographic area of Utah. Results associated with photos at BYU may have confounding factors (including comfort/familiarity with campus) as all participants were current BYU students. As this study was an initial explorative step, the next ones should include a broader sampling strategy, including follow-up with participants to discuss what they saw, and why.

## Conclusions

Our study demonstrates point-of-view differences in female–male walking space assessments. Overall, women focused on areas outside the path more often than men, for example, Figures 2 and 3, which may be reflective of their broad experience with fear of crime that highlights a greater fear of crime, particularly personally violent or sexual crimes resulting from stalking, prowling, or loitering in poorly lit areas. Despite attempts to improve environment, such as lighting, it is likely these findings represent a more systematic problem, rippling into other areas of women's lives. The results presented here can be a useful conversation starter for recognizing different lived experiences and to begin reclaiming everyday spaces for free mobility (Beebeejaun, 2017). Viewing walking safety through the lens of lived experience could be productive in terms of holistically building community trust and shared responsibility for ourselves and others in supporting the holistic safety and wellbeing of walk commuters.

## Data Access

Data for this study cannot be shared because participants did not consent to public data sharing.
